# An iterative algorithm for optimizing COVID-19 vaccination strategies considering unknown supply

**DOI:** 10.1371/journal.pone.0265957

**Published:** 2022-05-02

**Authors:** Martin Bicher, Claire Rippinger, Melanie Zechmeister, Beate Jahn, Gaby Sroczynski, Nikolai Mühlberger, Julia Santamaria-Navarro, Christoph Urach, Dominik Brunmeir, Uwe Siebert, Niki Popper

**Affiliations:** 1 Institute for Information Systems Engineering, TU Wien, Vienna, Austria; 2 dwh Simulation Services, dwh GmbH, Vienna, Austria; 3 DEXHELPP, Association for Decision Support for Health Policy and Planning, Vienna, Austria; 4 Department of Public Health, Health Services Research and Health Technology Assessment, Institute of Public Health, Medical Decision Making and Health Technology Assessment, UMIT–University for Health Sciences, Medical Informatics and Technology, Hall in Tirol, Austria; 5 Institute for Technology Assessment and Department of Radiology, Massachusetts General Hospital, Harvard Medical School, Boston, MA, United States of America; 6 Center for Health Decision Science, Departments of Epidemiology and Health Policy & Management, Harvard T.H. Chan School of Public Health, Boston, MA, United States of America; University of Hong Kong, HONG KONG

## Abstract

**Background and objective:**

The distribution of the newly developed vaccines presents a great challenge in the ongoing SARS-CoV-2 pandemic. Policy makers must decide which subgroups should be vaccinated first to minimize the negative consequences of the pandemic. These decisions must be made upfront and under uncertainty regarding the amount of vaccine doses available at a given time. The objective of the present work was to develop an iterative optimization algorithm, which provides a prioritization order of predefined subgroups. The results of this algorithm should be optimal but also robust with respect to potentially limited vaccine supply.

**Methods:**

We present an optimization meta-heuristic which can be used in a classic simulation-optimization setting with a simulation model in a feedback loop. The meta-heuristic can be applied in combination with any epidemiological simulation model capable of depicting the effects of vaccine distribution to the modeled population, accepts a vaccine prioritization plan in a certain notation as input, and generates decision making relevant variables such as COVID-19 caused deaths or hospitalizations as output. We finally demonstrate the mechanics of the algorithm presenting the results of a case study performed with an epidemiological agent-based model.

**Results:**

We show that the developed method generates a highly robust vaccination prioritization plan which is proven to fulfill an elegant supremacy criterion: the plan is equally optimal for any quantity of vaccine doses available. The algorithm was tested on a case study in the Austrian context and it generated a vaccination plan prioritization favoring individuals age 65+, followed by vulnerable groups, to minimize COVID-19 related burden.

**Discussion:**

The results of the case study coincide with the international policy recommendations which strengthen the applicability of the approach. We conclude that the path-dependent optimum optimum provided by the algorithm is well suited for real world applications, in which decision makers need to develop strategies upfront under high levels of uncertainty.

## 1 Introduction

By summer 2020, medical trials showed that the first candidates for vaccines are effective against COVID-19. At this time, countries worldwide needed to prepare the start and logistics of the vaccination process, requiring many decisions under uncertainty. A key decision at that time was to define which population groups should be vaccinated first under limited supply in order to be most efficient in terms of reducing the COVID-19 related burden.

The key element of this decision is that different person groups play different roles in the epidemic: While, for example, young adults can be considered as driving forces of the disease spread due to their increased number of daily contacts (see [1]), elderly are more vulnerable to suffer severe disease progressions. Consequently, prioritization of either of the groups would reduce the burden of disease in a direct (i.e. vaccination of risk groups) or indirect (i.e. reducing the disease prevalence) way.

Considering the complexity of the dynamics between vaccinations, transmissions, and severe, critical, and fatal cases, modeling and simulation is the only possibility to evaluate and compare different vaccination prioritization strategies upfront. Therefore, it is necessary to develop and validate a simulation model that validly depicts these nonlinear dynamics and furthermore apply the model to compare different vaccination strategies.

Since we cannot simply try out every possible vaccination strategy—the “parameter space” is too large—a heuristic must be defined, which systematically generates and evaluates plans, given a certain optimization target measure, e.g. deceased COVID-19 cases. Defining such an algorithm is challenging, since (1) the parameter space must be defined, understood, formulated and intelligently searched, and (2) the result should not only try to optimize a certain target value, but should also be robust to limited vaccine supply.

There are already several studies on the subject of optimal vaccine distribution for SARS-CoV-2 or other infectious diseases such as influenza. While some of them limit their findings on evaluating several previously fixed strategies [2], others use different optimization algorithms to find the best solution in a given parameter space [3, 4]. In [5], the vaccination prioritization problem is tackled with a network-approach and a Mixed Linear Integer Problem, yet the outcomes are complicated to interpret in a policy context, since the focus is to determine super-spreaders. In [6], prioritization concepts from influenza are re-highlighted in the COVID-19 context.

Anyway, neither of the cited approaches, nor any of those reviewed in [7], explicitly incorporated the uncertainty regarding the stream of doses available over time. In [8], the authors consider limited supply since the question is tackled from an economical point of view, yet no epidemiological concepts are included. In [9], epidemiological aspects are considered, yet no prioritization of subgroups are discussed.

Our team has developed an optimization algorithm which satisfies the stated requirements. To underline the real-world applicability of the concept, the strategy has already been applied for counseling the Austrian vaccination strategy planners in Autumn 2020. The results of this case study have already been published in [10]. In this work, we present the methodological details of the optimization algorithm, which by itself develops a SARS-CoV-2 vaccination strategy using a simulation model in a simulation-optimization feedback loop:

1. The algorithm specifies the input to a simulation model in the form of an abstract vaccination-plan.
2. The model is executed and the output of the model is fed back to the algorithm.
3. According to the simulation results, the algorithm adapts the input with the goal to optimize a target function. The process continues with step 1 until a termination criterion is met.

We show that our algorithm is not only comprehensible, flexible and efficient, but also provides a path-dependent optimum of the solution which guarantees that the prioritization is robust against interruptions, for example if vaccine shortages occur. Finally, we will present results from analyses performed in the Austrian context.

## 2 Methods

In the following, we will explain the optimization meta-heuristic formally in several steps. First, we specify the necessary requirements on the simulation model and discuss suitable target variables. In a second step, we define, how a vaccine prioritization strategy is defined formally and introduce a specific notation for it. Furthermore, we properly present the developed meta-heuristic and how it is used in the simulation-optimization setup. Finally, potentially occurring problems and corresponding solutions are discussed. In the last section of the Methods part, we give a brief summary of the case study and of the applied simulation model.

### 2.1 Simulation model, target variables and baseline scenario

In order to evaluate vaccination prioritization strategies, a simulation model must be provided, which is capable of estimating the impact of the strategy. The model must be able to depict the spread of the disease, the vaccination processes as well as the impact of the latter on the prior. Thus, the model must be capable of distributing a certain number of vaccine doses to a specific sub population at a given point in time—we will furthermore call these *Vaccination Events*. Each event is specified by an event-time, a number of available doses *d* and a target group *G* that specifies, which persons are entitled to get one of the doses. We term the combined information (*d*, *G*) of doses and target group *batch*.

A sequence of batches and event-times must serve as the model’s input. The model output must be suitable as target variable of the optimization algorithm (see below). In principle, any scalar outcome variable of the model that quantifies the burden of morbidity would be a suitable target for the optimization algorithm, for example the cumulative number of infections, hospitalizations or deaths.

Finally, a proper definition of a baseline scenario of the simulation is crucial. Although the vaccinations themselves will influence the epidemic spread, the effect of the vaccinations will be influenced by the underlying epidemic situation as well. This includes, in particular, whether or not any policies besides vaccination are active to contain the disease, and the value of *R*_*eff*_ by the time of the first vaccinations. The choice of the baseline scenario strongly depends on the research question of the case study.

### 2.2 Prioritization strategy concept

Before a prioritization strategy can be established, it is necessary to define target groups. There are various ways how such groups can be defined. The most common ones include

- grouping by age,
- grouping by vulnerability (hypertension, obesity, …),
- grouping by environmental setting (household member of school children, household member of a pregnant person, …)
- grouping by profession (health care worker, teacher, police officer,…),

as well as combinations of these properties. The rationales for defining these groups typically depend on the vaccination strategy goal but can also be politically or logistically motivated.

To tackle this concept technically, we define

$$\bar{G}≔\left\{G_{j},j\in \left\{1,\ldots ,n\right\}\right\}$$

as the set of groups we want to consider in our vaccination plan. Each group *G*_*j*_ is a subset of the population and consists of those individuals that share a certain property *p*_*j*_. Note, that ⋃_*j*_ *G*_*j*_ can also be a real subset of the population, since there might also be persons who are not eligible to be vaccinated (e.g. the vaccine is not approved for them). Moreover, the individual groups must not necessarily be disjoint.

### 2.3 Batch-notation and iterative optimization algorithm

In any classic simulation-optimization procedure, two interfaces between optimization routine and simulation model are necessary, one for the output of the optimization algorithm and the input to the simulation, and one for the output of the model and the input of the optimization algorithm. In the present case, the prior is more challenging since the algorithm must “tell” the simulation model, how to vaccinate the agents.

Our solution to this problem is based on the concept of vaccination *batches*, characterized by size *d* and target group *G* (see Section 2.1). Goal of the iterative strategy is to find a sequence of batches, which minimizes the output variable in a precise sense, which we will discuss later.

To properly describe the algorithm, we will use a specific notation, furthermore called *batch-notation*:

$$\begin{array}{c} x_{k}=(\left(d_{1},G_{j_{1}}\right),\left(d_{2},G_{j_{2}}\right),\ldots ,\left(d_{k},G_{j_{k}}\right)) \end{array}$$ (1)

where (*d*_*i*_)_*i*∈{1,…,*k*}_ denotes a vector of batch sizes and ${\left(G_{j_{i}}\right)}_{j_{i}\in \left\{1,\ldots ,n\right\}}$ a vector of target groups.

We will use the introduced *batch-notation* for three purposes:

- Any *x*_*k*_ can directly be interpreted as a vaccination prioritization plan with *k* batches and $\sum_{i=1}^{k}d_{i}$ total doses.
- Second, the model is adjusted to use elements *x*_*k*_ as model input. The simulator creates *k* *Vaccination Events* at predefined times *t*_*i*_, *i* = 0 … *k*. In general, *t*_1_ ≤ *t*_2_ ≤ … ≤ *t*_*k*_ makes sense, indicating that batches are delivered bit by bit.
- Third, we are able to formulate the optimization goal and the iterative algorithm.

To simplify the formal specification, we define *f*(*x*) as the function that maps the vaccination prioritization strategy *x*, given in batch-notation, onto the investigated target variable of the simulation $f\left(x\right)\in R^{+}$. That means,

$$\begin{array}{c} f=(\text{evaluate}\;\text{target}\;\text{variable})\circ (\text{interpret}\;\text{x}\;\text{as}\;\text{input}). \end{array}$$

A strategy *x* is furthermore said to be superior to a competing strategy *x*′ if *f*(*x*) < *f*(*x*′).

Thus, the iterative algorithm results as follows:

1. We initialize the algorithm with an empty state *x*_0_ = ().
2. Given a total of *k*_*tot*_ batches with ${\left(d_{j}\right)}_{j=1\ldots k_{tot}}$ doses for each batch, the optimization algorithm performs *k*_*tot*_ steps. In each step *k*:
  1. For the current state *x*_*k*_, we create *n* different enhancements ${\left(x_{k}^{\prime }\right)}^{j}$ for *j* ∈ 1, …, *n* by adding a new batch assigned to *G*_*j*_ to *x*_*k*_. Thus ${\left(x_{k}^{\prime }\right)}^{j}=(\left(d_{1},G_{j_{1}}\right),\left(d_{2},G_{j_{2}}\right),\ldots ,\left(d_{k},G_{j_{k}}\right),\left(d_{k+1},G_{j}\right))$
  2. For each of the *n* new states created, we evaluate $f(x_{k}^{\prime })$ using the corresponding state $x_{k}^{\prime }$ as model input.
  3. We compare the *n* outcomes and assign $x_{k+1}\leftarrow {\left(x_{k}^{\prime }\right)}^{l}$ with $l={\text{arg min}}_{i=1\ldots n}\left(f{\left(x_{k}^{\prime }\right)}^{i}\right)$ as the new state of the algorithm.

**Fig 1:**
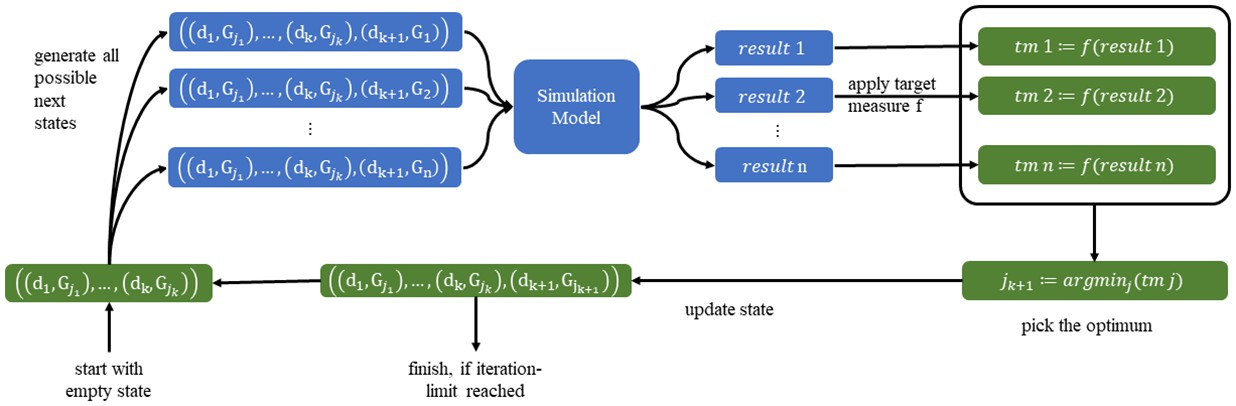
Iterative algorithm to optimize the distribution of the *batches*. The algorithm is started with an empty state and is terminated as soon as the sum of all state-exponents matches a predefined maximum iteration number.

This algorithm, also shown schematically in Fig 1, is simple and has many useful features, which we will elaborate in the Discussion. Its final state $x_{k_{tot}}$ is the output of the algorithm and can be interpreted as optimized prioritization strategy.

### 2.4 Invalid plans and parts of batches

Both, in reality and in the model, the *batch-notation* might create unfeasible vaccination plans because an assigned batch is too large for its target group. Usually, this is the case if many members of the group are already vaccinated in previous batches.

To apply the algorithm, the simulation model is required to feedback this information. Say, $\tilde{d}$ defines the number of entitled persons in the target group in the model and *d*_*k*_ is the originally scheduled size of the batch. In case $\tilde{d}<d_{k}$, the model will feedback $\tilde{d}$ instead of generating a result. Moreover, two cases will be distinguished:

- In case $\tilde{d}=0$, the vaccination plan is considered invalid. The corresponding batch tuple is not regarded any further.
- In case $0<\tilde{d}<d_{k}$, the batch is split to ensure that no doses are wasted.

For the second case, two possible strategies can be applied in the optimization algorithm:

For the first strategy, all computations for the *k*-th batch are stopped immediately. The total number of batches *k*_*tot*_ is increased by one and the vector of batch sizes is redefined to

$${\left(d_{j}\right)}_{j=1\ldots k_{tot}}=d_{1},\ldots ,d_{k-1},\tilde{d},\tilde{d}-d_{k},d_{k+1},\ldots ,d_{k_{tot}}.$$

Furthermore, iteration *k* is restarted with the adapted batch size.

For the second strategy, only the simulation which produced the invalid outcome is restarted with a different setup. For this setup, $\tilde{d}$ doses are distributed as planned while the remaining $\left(d_{k}-\tilde{d}\right)$ doses are assigned to a predefined alternative group. Therefore, it is most convenient to define a fixed a-priori order, say, *G*_1_ > *G*_2_ > …*G*_*n*_, and choose the first group in this list that leads to a valid vaccination plan.

Clearly, strategy two comes with smaller computation times, but has flaws with respect to producing the expected optimal algorithm outcome. Nevertheless, errors can be expected to be comparably small, if batch sizes are a-priori chosen small as well.

### 2.5 Case study

The algorithm was developed in the context of counseling Austrian decision makers in mid 2020 during the COVID-19 pandemic. We will briefly describe the underlying simulation model, as well as the setup of the optimization algorithm, such as target groups, batch sizes, target variables and the epidemiological baseline scenario of the simulation. A comprehensive description of the case study can be found in [10].

#### 2.5.1 Disease model

We use an agent-based model mainly developed in spring 2020 to simulate the spread of SARS-CoV-2 in Austria. This model has been subject to multiple studies (see [11, 12]) and is actively used as a decision support tool for the Austrian COVID-19 containment policies [13].

In this model, every member of the Austrian population is modeled by a statistical representative based on age, sex and place of residence. The agents form a contact network consisting of household-, work-, school-, and leisure-time-contacts, representing the social behavior of the Austrian population [1]. Furthermore, person-to-person-contacts in this network spread the virus. Once an agent is infected, a detailed case/patient pathway is initialized, based on events. After a sampled latency period, the agent becomes infectious and has the potential to develop symptoms of COVID-19 and might require treatment. People with mild or no symptoms can recover at home, whereas people with severe or critical symptoms require admission to a hospital or an intensive care unit (ICU), respectively. All risk factors, including the risk for subsequent death from COVID-19, depend on age and medical history of a person.

The vaccination of an individual is modeled as a single event with immediate effect on the person’s susceptibility. The effectiveness of the vaccine is modeled by a Bernoulli process with an age-dependent probability that decides whether the vaccination was effective or not. At each vaccination event, the list of all model agents is first filtered for entitlement: Agents must neither be actively confirmed SARS-CoV-2 cases nor have already received a vaccine dose before and they must be part of the selected target group *G*. In a second step, a subset with the size of the *batch* is randomly selected and vaccinated, according to the model logic explained above:

$$\begin{array}{c} \text{agents}⊇\text{non-confirmed,}\;\text{non-vaccinated}⊇\text{member}\;\text{of}\;G⊇d\;\text{randomly}\;\text{chosen} \end{array}$$

The agent-based disease transmission model allows to observe a multitude of different outcomes. The ones most relevant to this study are confirmed infected, hospitalized, ICU-hospitalized and deceased agents. These numbers are carried out for each time step (daily) and are available via different counting methods: active, new and cumulative.

A comprehensive model description including a list of all parameter values used can be found in the supplemental material of [14].

#### 2.5.2 Target variables and baseline scenario

We defined the cumulative number of COVID-19 caused deaths as our main target variable. We accumulated the cases in a simulated time frame of six months after vaccination start, taking the average of 10 Monte-Carlo runs.

As the baseline scenario, we investigated a scenario informally called “fictional wave”: the outbreak of the disease is only damped by the performed vaccinations and, of course, natural herd immunity. Although this scenario is entirely fictional—it does not regard any policies against the spread or changed human behavior—it is easy to explain and free of bias. Even though this scenario cannot directly be compared to the real development, a comparison between different vaccination strategies is well possible.

A simulated time span of six months from January 1st to July 1st was found sufficient to cover a full epidemic wave from outbreak to (disease induced) herd immunity.

#### 2.5.3 Target groups and vaccination batches

In total, five different target groups were defined in the case study after workshops with clinical experts and decision makers. Three of these groups are age-based, classifying persons as *young* (*Y*, 15—44 years), *middle-aged* (*M*, 45—64 years) or *elderly* (*E*, 65+ years). The fourth group consists of *vulnerable* (*V*) people with a higher risk for a severe or critical course of COVID-19 due to comorbidity. Finally, the last target group in our case study consists of *health care workers* (*H*), that is, medical staff in hospitals and outpatient care, medical practitioners, mobile care, long-term care facilities, etc. Agents are classified as health care workers according to the age-distribution of these professions in Austria. We will refer to these groups as *G*_*Y*_, *G*_*M*_, *G*_*E*_, *G*_*V*_ and *G*_*H*_ accordingly. Since by the time of the study, no COVID-19 vaccine has been licensed for children, most children below 15 are not part of any of the groups. Moreover, groups *G*_*E*_ and *G*_*V*_ are strong overlapping.

Since the simulation model comes with long computation times and involved decision makers needed quick results, we chose strategy two with respect to dealing with invalid vaccination plans (see Section 2.4). In collaboration with the experts, we defined the a-priori prioritization *G*_*E*_ > *G*_*V*_ > *G*_*H*_ > *G*_*M*_ > *G*_*Y*_, which would be used if a vaccination plan results in leftover doses.

Four batches—the first with size 250 000, the others with size 750 000—were considered. Since no information about vaccine deliveries has been available by the time of the study, we used *t*_0_ = *t*_1_ = *t*_2_ = *t*_3_ = January 1st. It is plausible to assume, that different choices for *t*_*i*_ would only change the magnitude of the outcome variables, but would lead to the same priorities, since the baseline scenario is a homogeneous epidemic wave.

## 3 Results

By definition of the strategy, the iterative optimization algorithm provides a dynamic optimum. Formally this is interpreted as follows: given the final result $x_{k_{tot}}=i_{1}^{k_{1}}\ldots i_{m}^{k_{m}}$ of the algorithm, any sub-vector of this result *x*_*k*_, *k* ≤ *k*_*tot*_ is chosen optimal w.r.t. a given *x*_*k*−1_:

$$x_{k}={\text{argmin}}_{f\left(y_{k}\right):y_{k-1}=x_{k-1}}\left(f\left(y_{k}\right)\right)$$

That means, if the actual numbers of vaccine batches is not precisely known in advance, this strategy provides the most robust solution. Nevertheless, a global optimum

$${\text{argmin}}_{y_{k_{tot}}}\left(f\left(y_{k_{tot}}\right)\right),$$

is not necessarily provided.

To better illustrate the iterative nature of the optimization algorithm, we show selected interim simulation results which have not been presented in the case study [10].

**Fig 2:**
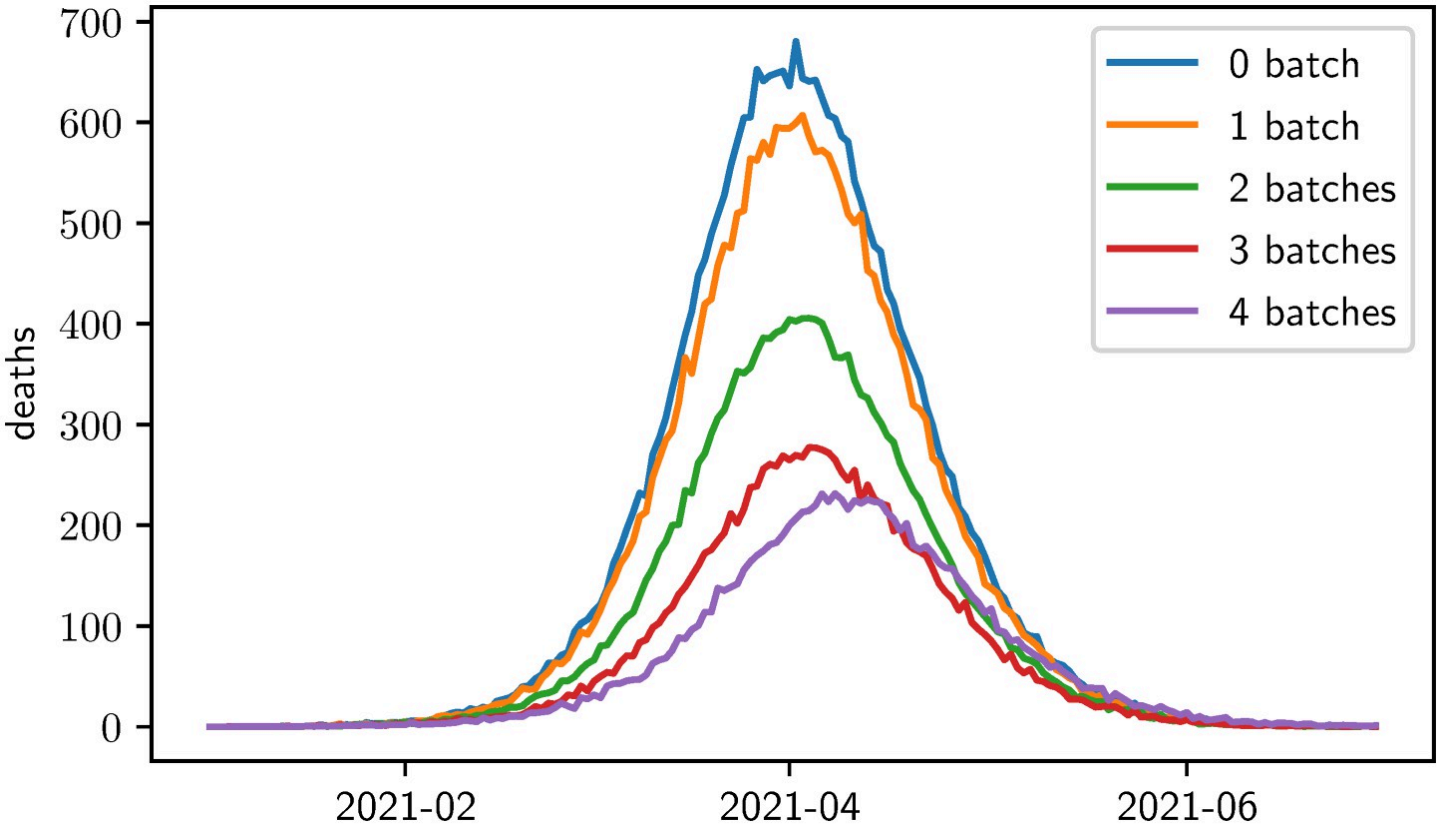
Model results for daily COVID-19 caused deaths after every step of the iterative optimization algorithm. In each step, one additional batch has been distributed. The target variable for the optimization algorithm is the number of cumulative COVID-19 caused deaths.

**Fig 3:**
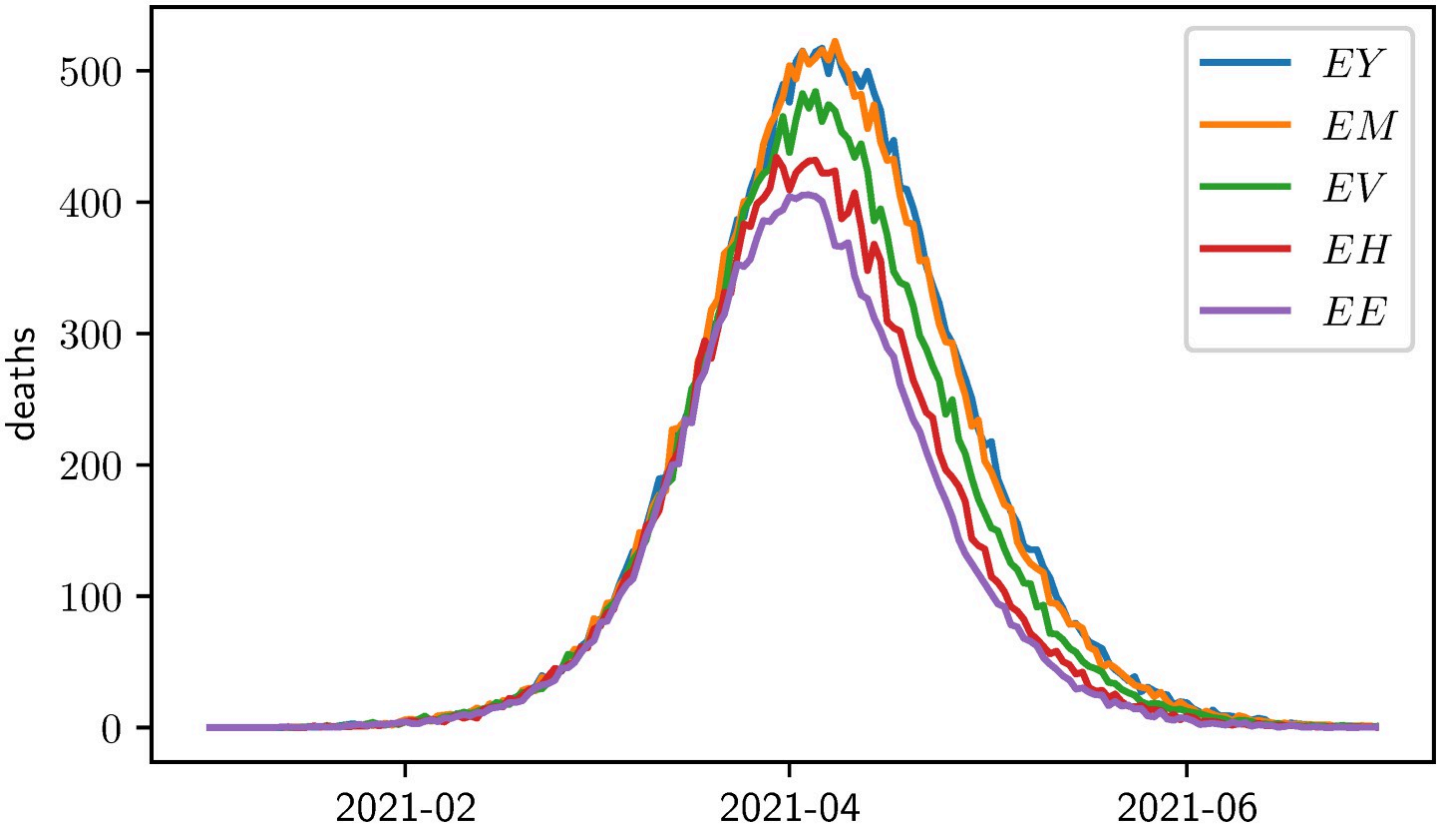
Model results for daily COVID-19 caused deaths for simulations performed during the second step of the optimization algorithm. Each line represents one of the enhancements ${\left(x_{1}^{\prime }\right)}^{i},i\in \left\{Y,M,E,V,H\right\}$ with *x*_1_ = *E*. The target variable for the optimization algorithm is the number of cumulative COVID-19 caused deaths.

**Fig 4:**
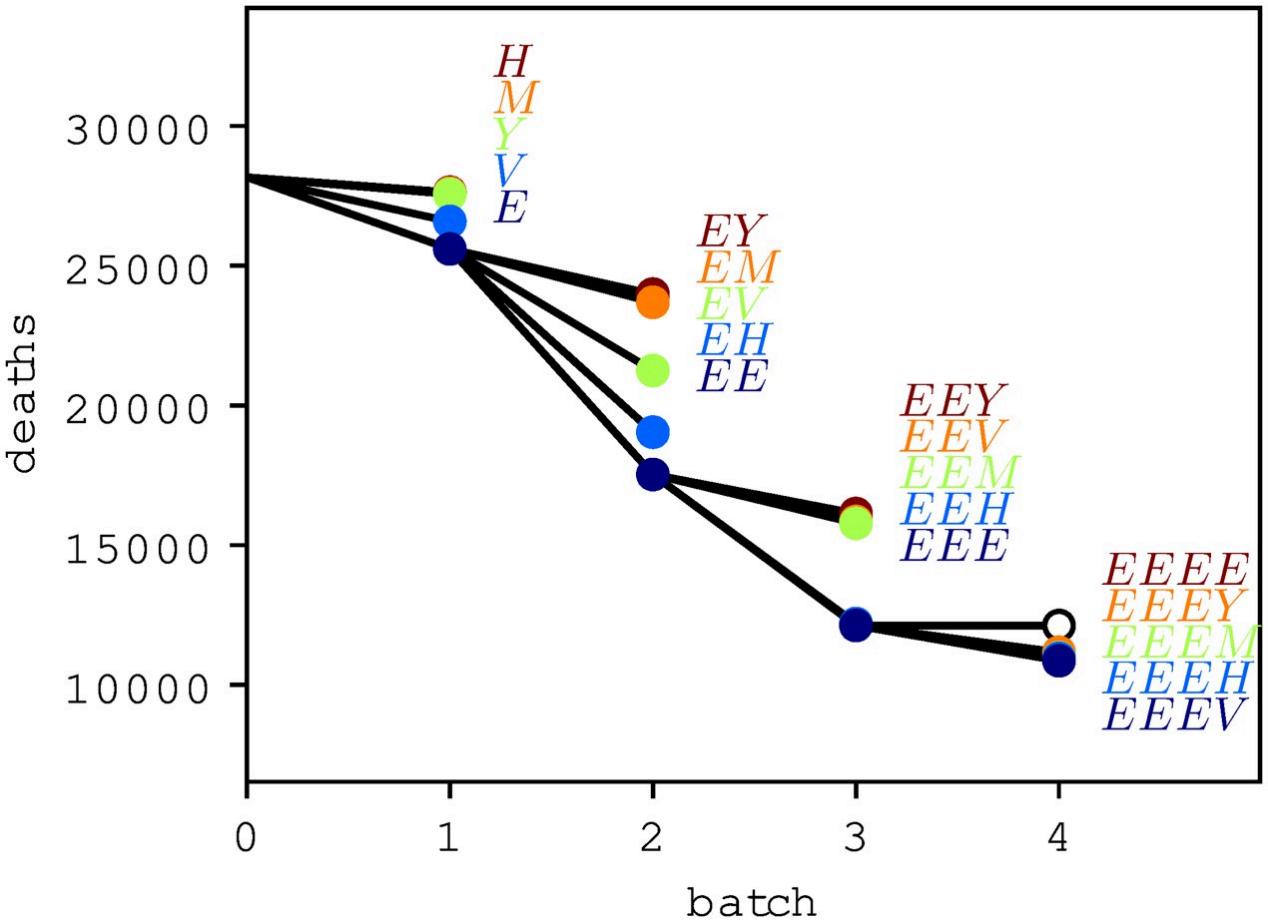
Accumulated model results for daily COVID-19 caused deaths for all the simulations performed during the iterative optimization. After each step / batch, the simulation enhances the batch-notation that yielded the best results for the target variable. In the last iteration, *EEEE* led to an invalid plan since the corresponding group *G*_*E*_ is already fully vaccinated after *EEE*.

Fig 2 gives an overview of the timeline of the COVID-19 caused deaths after each of the 4 steps of the optimization algorithm. Clearly, every additional batch reduces this optimization target variable. To update the state, another 5 simulations are executed at each step, one for each person group. Fig 3 shows the corresponding five results within the second step of the optimization algorithm. After the first step, it holds that *x*_1_ = (*d*_1_, *G*_*E*_) = *E*. In the simulations performed during the second step of the optimization algorithm, the lowest number of COVID-19 caused deaths is achieved if the second vaccination batch is distributed to the elderly and thus *x*_2_ = ((*d*_1_, *G*_*E*_), (*d*_2_, *G*_*E*_)) = *EE* will be the next state of the algorithm. Fig 4 finally displays the development of the target variable (cumulated COVID-19 caused deaths) over all 4 ⋅ 5 = 20 simulated strategies. The black lines indicate how the algorithm enhanced the vaccination strategy iteratively.

The source data is available online under https://git.dwh.at/mlandsiedl/Vaccination-Strategy-Optimizer-Data

## 4 Discussion

In this work, we presented an algorithm for early stage development of a vaccination prioritization plan. The algorithm applies a dynamic simulation model in the loop and converges to a vaccination strategy, which can be recommended to decision makers. The result of the algorithm fulfills a path-dependent optimum, which is well suited for being applied in early stage planning, if the expected number of available vaccination doses is not well known in advance or shortages are to be expected. In this particular research question, this version of optimum is usually superior to a global optimum, since vaccine production and deliveries are associated with a high level of uncertainty.

This superiority can be shown using a simple thought experiment: Considering a sterilizing vaccine and a base reproduction rate of about three, which corresponds to standard estimates for the original SARS-CoV-2 virus, it is plausible that vaccinating all inhabitants between 16 and 55 (about 55% of the Austrian population) will cause *R*_*eff*_ to drop below 1, since these population groups are responsible for most (infectious) contacts. In this scenario, also the elderly, which are most endangered to have severe disease progressions, are well protected by declining case numbers. In case of a shortage with only half of these doses available, the spread of the disease cannot be stopped by the vaccines and the numbers stay high. In this scenario, elderly persons are not protected at all—neither by vaccination nor by dropping case numbers. The vaccine plan was a failure and thousands of lives were put at risk.

Consequently, the algorithm provides a stable vaccination plan that is equally optimal, if interrupted in the middle.

Moreover, the algorithm executes with linear increasing computational effort—that is *k*_*tot*_ ⋅ *n* simulation runs (Monte Carlo simulations excluded). Finding a global maximum, in the worst case, would require $n^{k_{tot}}$ simulation runs.

The performed case study in summer 2020 demonstrated, that the strategy works well and is flexible. Note, that even in this deliberately simplified case study, which was coordinated with important health care experts and stake holders in Austria, many of the carefully described “special cases” occurred: First, the person groups are not disjoint since *G*_*H*_, *G*_*V*_ and *G*_*E*/*M*/*Y*_ overlap. Moreover, the union of all groups does not cover the whole population, since children were not included in the vaccination program by this time. Finally, batches had to be differently sized (forced prioritization of health-care workers) and sometimes needed to be split and distributed on two groups.

Due to the proper definition of the algorithm, the results of the analysis could be used for counseling the Austrian vaccination strategy planners [10]. Moreover, the results have been strengthened by recommendations of many other institutions including WHO [15], CDC [16] and ECDC [17], which come to similar conclusions.

In addition to COVID-19 caused deaths, we also investigated cumulative SARS-CoV-2 confirmed cases and intensive care (ICU) patients as target variables of the algorithm. Interestingly, different target measures led to different prioritization plans. The main reason for this lies in the model mechanisms for state-transitions between case, confirmed case, hospitalized/ICU patient and deceased case, all of which are highly age-dependent. This feature resulted in the interesting result, that deceased and ICU patients can be reduced best directly, by vaccination of the corresponding cohorts, whereas (confirmed) cases can be reduced best indirectly, by vaccination of the cohorts with most contacts. See [10] for more details.

One limitation of the presented optimization algorithm is the inability to provide a global optimium at the price of escape strategies and computation time. This means, if the precise number of doses that will become available for vaccination is well known in advance, the proposed algorithm is not the best choice and a different optimization must be applied—e.g. either a full grid-search or some metaheuristic method. Since the SARS-CoV-2 vaccination situation has been unclear by the time of the vaccination plan design in Austria, the strategy was well suited in our case.

In the past year, the presented method and the performed case-study played a key-role in preparing the Austrian decision makers for the upcoming SARS-CoV-2 vaccination campaign in spring 2021 [10]. By mid 2021, the campaign shows first effects on the case numbers and even more on hospitalizations and deaths. Yet, still the danger of new mutations, comparable to infamous lineage B.1.617.2 for which current vaccines are estimated to have reduced effectiveness [18], is omnipresent. As a result, pharmaceutical companies work hard on refined vaccines for which new vaccination prioritization plans might become necessary in near future.
